# Supplementary material for: Strong room-temperature bulk nonlinear Hall effect in a spin-valley locked Dirac material
Source: Nat Commun. 2023 Jan 23;14:364. doi: 10.1038/s41467-023-35989-0 (PMC9871029; doi:10.1038/s41467-023-35989-0)
Supplement: Supplementary file 1 — Supplementary information [file 41467_2023_35989_MOESM1_ESM.pdf]

SUPPLEMENTARY INFORMATION

Strong Room-Temperature Bulk Nonlinear Hall Effect in a Spin-Valley Locked Dirac Material

Lujin Min[1,2], Hengxin Tan[3], Zhijian Xie[4], Leixin Miao[2], Ruoxi Zhang[1], Seng Huat Lee[1,5], Venkatraman Gopalan[2], Chaoxing Liu[1], Nasim Alem[2], Binghai Yan[3]*, Zhiqiang Mao[1]*
[1]Department of Physics, Pennsylvania State University; University Park, PA, USA
[2]Department of Materials Science and Engineering, Pennsylvania State University; University Park, PA, USA
[3]Department of Condensed Matter Physics, Weizmann Institute of Science; Rehovot, Israel
[4]Department of Electrical and Computer Engineering, North Carolina Agriculture &Technical State University; Greensboro, NC, USA
[5]2D Crystal Consortium, Materials Research Institute, Pennsylvania State University; University Park, PA, USA
* Corresponding author. Email: binghai.yan@weizmann.ac.il (B.Y.); zim1@psu.edu (Z.M.)

Contents:

Supplementary Notes 1-10
Supplementary Figs. 1-14
Supplementary Table 1
Reference

Supplementary Notes

1. Sample information

In Supplementary Fig. 6, we present the room-temperature normal Hall effect measurement results for all the devices studied in this work. All of them exhibit n-type transport behavior. We estimated the carrier densities of these samples at 10K, room temperature (300K), and 350K from the measured Hall coefficients and list them in Table S1.

2. Determination of crystallographic axes and domain structures of $BaMnSb_2$

Since the orthorhombic distortion of $BaMnSb_2$ is small and hard to be resolved by diffraction techniques such as neutron scattering, X-ray diffraction, or electron backscatter diffraction, we determined the crystallographic axes of the samples used in this study using transmission electron microscopy (TEM) and scanning transmission electron microscopy (STEM). From the crystal used for fabricating the HB2 device, we took a cross-sectional lamella sample across the 90° domain wall through focused ion beam (FIB) cutting (see Supplementary Figs. 3a & 7a). Fig. S7b shows the dark field (DF) TEM image taken from the specimen, acquired using the g = 020/200 reflection. The 90° domain wall observed under the polarized microscope is visible in the middle of the sample, dividing the specimen into *a*-axis oriented domain (left) and *b*-axis oriented domain (right). Notably, the 90° domain wall is vertical and straight near the surface of the sample, whereas it is bent to the right as the depth increases, indicating that the domain structures beneath the surface could be different from the surface domain distribution observed using an optical microscope. Additionally, numerous domains in the *b*-domain were spotted due to the alternating bright-dark contrast in the DF-TEM image, which is proved to be 180° domains, as discussed below.

Supplementary Figs. 7c&d present the atomically resolved annular dark field (ADF-) STEM images taken, respectively, at the approximate positions indicated by the red and cyan spots on the *a*-domain and *b*-domain shown in Supplementary Fig. 7b. The insets are the magnified images with the crystal model overlays. The previous study[1] determined that the Sb columns within the 2D Sb zig-zag chain layers are evenly spaced when viewed from the [100] zone axis, while the Sb columns shift and form 'dumbbell-shaped' pairs when viewed from the [010] zone axis. Based on the shift of the Sb columns, we determined the zone axes to be [100] and [010] in the *a*-domain and *b*-domain, respectively, as shown by the crystal model overlays. Based on that, we labeled the crystallographic axis of sample HB2 in the inset of Supplementary Fig. 3a.

Given that the orientation of the crystallographic axis of sample HB2 is known, we could determine the crystallographic orientations of other lamellar crystals using HB2 as a reference through polarized microscopy. Under the same polarization configuration, the domains with the same color should share the same crystallographic orientation. To double-check this method, another FIB lamella sample was extracted at the center of the crystal which was used for fabricating HB1, as shown in Supplementary Fig. 8a. The ADF-STEM image of this specimen (Supplementary Fig. 8b) clearly shows the Sb 'dumbbell-shaped' pairs, indicating the *b*-axis is perpendicular to this lamellar sample, which is consistent with the crystallographic axis determined based on the color contrast of HB2. As stated in the main text, the crystallographic axes of samples C1-L & C1-R were not determined. This is because we did not realize that we could use STEM to determine crystallographic directions until we finished measurements on C1-L & C1-R. We determined the crystallographic axes for all other samples used in this study using the method discussed above, including C2, C3, and C4.

In addition, we observed the 180° domain walls from the atomically resolved STEM imaging and atomic displacement analysis[2], as shown in Supplementary Figs. 7b & 9a. The ADF-STEM image shown in Supplementary Fig. 9a, which was taken at the approximate position marked by the green dot in Supplementary Fig. 7b, shows a clear 180° domain wall. The red and cyan arrows superimposed on this STEM image represent the displacement vectors of the Sb columns along the [100] or [$\bar{1}$00] directions; the displacement vector map highlights the switching of the atomic displacement that occurs at the 180° domain wall (white dashed line). The profile of the displacements is shown in Supplementary Fig. 9b. Supplementary Figs. 9c&d show the refined Sb atomic column positions overlaid on the magnified STEM image from the yellow box from each domain in Supplementary Fig. 9a. There are three Sb atomic columns for each zig-zag chain layer in $BaMnSb_2$, and we positioned and identified the Sb atoms near the unit cell center and edges and marked their positions as red and blue dots. The reference center positions (green) are then determined with the averaged position of the Sb atoms at the edges (blue). Subsequently, the displacement vectors are calculated by comparing the deviation of the center Sb atoms (red) from the reference center (green), as shown in Supplementary Figs. 9e&f. We quantified the average displacement magnitude of the Sb column to be 41.5 pm with a standard deviation of 7.1 pm, which is close to the reported value of 54.2 pm from the first principle calculation[1]. On the other hand, when observing the *a*-domain from the [100] zone axis, the Sb atoms are always evenly distributed, so there is no contrast between the two sides of the 180° domain wall, and thus 180° domains can only be observed in the *b*-domain (Supplementary Fig. S7b).

3. <u>Effect of 90° and 180° domains on the NLHE in $BaMnSb_2$</u>

For a given sample composed of mixed b-axis oriented and a-axis oriented domains (denoted by b-domain and a-domain respectively), when a current flows along the *b*-axis of the b-domains, the b-axis of a-domains would be perpendicular to the current, thus resulting in suppression of the nonlinear Hall voltage, as the a-domains do not generate any nonlinear Hall response. Besides the 90° domains, the 180° domains, which cannot be directly observed on surface imaging by a polarized microscope, could significantly suppress the NLHE. Given that the local crystal structure is rotated by 180° across the 180° domain wall, the nonlinear Hall voltages generated by the two domains across a 180° domain wall would have the opposite signs and thus cancel out each other. As a result, crystals with high density 90° and 180° domains would have suppressed NLHE. Although we have revealed the characteristics of 90° and 180° domains of $BaMnSb_2$ through STEM experiments (Supplementary Note 2), we could not tell the difference in domain structures between different samples. Different domain structures, together with chemical potential variation between samples, lead the NLHE of $BaMnSb_2$ to be sample dependent, as discussed in the main text.

According to the DF-TEM image, the thickness of the 180° domains varies from tens nanometers to the sub-micron scale. If the samples are thin enough, like what we used here, the population ratio of these two different domains would deviate from 50:50, hence the measured NLHE will still be strong. However, when the sample is too thick and the 180° domain population ratio is close to 50:50, the NLHE would be strongly suppressed.

We quantitively determined the BCD from the measured $V_\perp^{2\omega}$ using $\mathbf{D} = \frac{2\hbar^2(\frac{\sigma c}{2})^3 V_\perp^{2\omega} W}{e^3\,\tau(I^{\omega})^2}$, where c is the out-of-plane lattice parameter, $\hbar$ is the reduced Planck's constant, σ is the 3D conductivity, and W is the effective width of the sample[3]. To minimize the influence of the extrinsic effect, we chose the peak value of $V_\perp^{2\omega}$ to conduct our calculation. The maximum BCD calculated

from the sample HB2 is 0.074 Å, one order of magnitude smaller than the theoretical prediction, as a result of the domain effect we discussed above.

It is worth mentioning that the chemical inhomogeneity of the sample can result in different Fermi levels between different domains. Consequently, we imagine a scenario where two kinds of 180° domains produce opposite NLHE and their maximal values occur at slightly different temperatures, thus resulting in a sign change in the NLH voltage with the variation of temperature. The sign change of $V_{\perp}^{2\omega}$ near 220K in sample C4 (Fig. 4c in the main text) can be understood in terms of this model.

As shown in Supplementary Fig. 10, we can fit the data of sample C4 to a sum of one positive and one negative Gaussian peaks with the same width which respectively represent the NLHE generated in two different kinds of domains. The curve of domain 1 shows a peak at 273K, while the curve of domain 2 shows a peak at 267K. The sum of these two peaks agrees well with the raw data. This result also explains why the peak of sample C4 is narrower compared to the peaks of other samples, as reflected in Fig. 4c in the main text. If we plot the temperature-dependent nonlinear Hall voltage of only one domain together with those of other samples, we find they have similar peak profiles. This proves that the sign change of $V_{\perp}^{2\omega}$ near 220K comes from 180° domains.

In fact, other samples also have 180° domains. However, as long as the chemical potentials of these domains are close to each other, the final temperature-dependent NLH signal will still be in single-peak shape without a significant sign change. As the samples we used in our experiments were small, for most of the samples, the inhomogeneity was not severe. That is why only sample S4 shows an obvious negative signal.

4. Rectification effect in the radio frequency range

Supplementary Fig. 4a shows the schematic of a cross-like sample with the input of the radio frequency signal and output of the DC signal measurement. In this experiment, the RF signal generator was connected to one pair of terminals of the Hall bar while the dc output is measured in the other pair of terminals. Due to impedance mismatch and contact resistance, the power applied to the intrinsic device is calculated based on the following formula,

$$P_d=P_0\cdot(1-|\Gamma(\omega)|^2)\cdot R_0/R$$

where P_d is the power applied to the intrinsic device, P_0 is the input power, $\Gamma(\omega)$ is measured reflection coefficients, R_0 is the sample resistance, and R is the total device resistance including contact resistance. Supplementary Fig. 4b shows the plot of output dc voltage versus signal frequency.

5. Relationship between the amplitude of second-harmonic voltage, rectification voltage, and DC-induced NLH voltage

Here, we explain why the measured rectification voltage and dc-induced NLH voltage are always larger than the measured second-harmonic voltage.

According to the theory[3], the nonlinear Hall current density can be expressed as $j^{\mathrm{NLH}} \propto sin^2(\omega t) = \frac{1}{2} - \frac{1}{2}\cos(2\omega t)$. Here the first and second terms represent the DC rectified and 2nd harmonic Hall current respectively. This indicates that the rectified Hall voltage $V_{\perp}^{RDC}$ should be the same as the peak value of the 2nd harmonic voltage $V_{\perp}^{2\omega,peak}$. However, the 2nd harmonic voltage measured by a lock-in amplifier is the root-main-square (RMS) voltage $V_{\perp}^{2\omega}$ which is equal to $V_{\perp}^{2\omega,peak}/\sqrt{2} = V_{\perp}^{\mathrm{RDC}}/\sqrt{2}$. This explains why our measured $V_{\perp}^{RDC}$ is about 1.41-1.44 times

larger than $V_\perp^{2\omega}$. The previously reported ratio of $V_\perp^{RDC}/V_\perp^{2\omega}$ = 1.41 for $Ce_3Bi_4Pd_3$ [4] should be of the same origin.

Moreover, when comparing the ac and dc induced NLHE, the dc driving current is always equal to the RMS value of the ac driving current. Therefore, the dc-induced nonlinear Hall voltage is equal to the rectified voltage driven by ac. This is exactly what we saw in experiments.

6. Ion beam surface damage

The FIB device fabrication will damage the materials owing to the energetic Ga^{2+} ions. At the normal operating energies, the mean free path of the ions in metals is on the atomic length scale, and typical effective stopping distances are in the 10-nm range.[5] Therefore, the ions and all their kinetic energy are absorbed merely in a thin surface layer.

In our FIB cut devices, ion beam damage should not be an important problem for the following reasons. Firstly, the damaged surface layers are always amorphous due to the incident of the Ga ions. The inversion symmetry of such amorphous layers is preserved, accordingly, they cannot generate any nonlinear Hall signal. Additionally, the thicknesses of the samples are several microns, much larger than those of the amorphous layers. Thirdly, the damaged amorphous layers are less conductive compared to the pristine crystals as the enhancement of the scattering rate, so the driving current prefers to flow through the undamaged part.

However, due to the ion beam damage, the surface amorphous layers of the devices are easier to be oxidized. This explains why the FIB fabricated devices are degraded in a few months.

7. Exclusion of the second-harmonic responses irrelevant to NLHE

Previous studies have shown that the second-harmonic signal can also be generated by extrinsic effects irrelevant to NLHE, such as the contact junction effect, asymmetric sample shape, and thermoelectric effect.[3] These extrinsic effects and the methods used to exclude them from intrinsic NLHE are discussed in detail in several papers[3, 6]. Contact junction effect can be excluded by the observations of linear *I-V* curves and non-frequency-dependent second-harmonic signals. All the samples studied in this work indeed exhibit linear *I-V* characteristics (see Supplementary Fig. 11) and their second-harmonic Hall voltages are not dependent on frequency (Figs. 2c-2e in the main text), thus excluding the junction effect. Other extrinsic effects can be ruled out by the dependence of the second-harmonic signal on carrier type and density, consistent data from many combinations of electrodes, and the highly Hall-dominated second-order signal, e.g. nearly 90° nonlinear Hall angle. In the main text, we have not only shown the striking current direction dependence of the second-harmonic Hall voltage for $BaMnSb_2$ (i.e. nonlinear Hall response is observed only when the driving current is applied to the b-axis), but also demonstrated the nonlinear Hall angle close to 90°. Furthermore, we also find the second-harmonic Hall voltages display non-monotonic temperature dependences and reach maxima near room temperature in several samples, which should not happen to any extrinsic effects, but agrees well with the calculated energy dependence of Berry curvature dipole. All these results indicate the nonlinear Hall response observed in $BaMnSb_2$ has an intrinsic origin, i.e. arising from the Berry curvature dipole of the spin-valley locked Dirac state.

8. Scaling analyses of the NLHE for $BaMnSb_2$

The scaling law of the NLHE has been theoretically proposed[7] and experimentally studied[6, 8, 9]. According to the previously reported works, the strength of the NLHE, $E_\perp^{2\omega}/\left(E_\parallel^{\omega}\right)^2$, monotonically increases with the increase of longitudinal conductivity, σ_{xx}, regardless of the

mechanism of the NLHE. In contrast, $E_{\perp}^{2\omega}/\left(E_{\parallel}^{\omega}\right)^2$ in $BaMnSb_2$ exhibits a non-monotonic dependence on σ_{xx}, as shown in Supplementary Fig. 12. This can be well understood in term of intrinsic NLHE, since the BCD of $BaMnSb_2$ also shows a peak at 23 meV above the conduction band edge as discussed in the main text.

9. Relationship between the NLH voltage, $E_{\perp}^{2\omega}/\left(E_{\parallel}^{\omega}\right)^2$, and NLH conductivity

The nonlinear Hall conductivity is described by $\sigma^{(2)} = -\sigma^{(1)}V_{\perp}^{(2)}L^2/\left(V_{\parallel}^2 W\right)$, where $\sigma^{(1)}$ is the linear conductivity, $V_{\perp}^{(2)}$ is the transverse second-order Hall voltage, $V_{\parallel}$ is the voltage of frequency ω along the longitudinal direction, and L and W are the length and the width of the channels, respectively.[10] Therefore, we can further convert it into $\sigma^{(2)} = -\sigma^{(1)}\,E_{\perp}^{2\omega}/\left(E_{\parallel}^{\omega}\right)^2$, where $E_{\perp}^{2\omega}$ and $E_{\parallel}^{\omega}$ represents transverse and longitudinal electric fields respectively and $E_{\perp}^{2\omega}/\left(E_{\parallel}^{\omega}\right)^2$ is proportional to the Berry curvature dipole **D**. **D** can be extracted via the equation $\mathbf{D} = \frac{2\hbar^2 {\sigma^{(1)}}^3 V_{\perp}^{(2)} W}{e^3 \tau \left(I_{\parallel}^{\omega}\right)^2}$, where τ is the scattering time which is proportional to $\sigma^{(1)}$.[3]

In Supplementary Fig. 13, we plot the temperature dependences of the normalized $V_{\perp}^{2\omega}$, $E_{\perp}^{2\omega}/\left(E_{\parallel}^{\omega}\right)^2$, $\sigma^{(1)}$, and $\sigma^{(2)}$ together for sample HB1. From 100K to 300K where we observed the nonlinear Hall effect, $\sigma^{(1)}$ shows a monotonic decrease with increasing temperature, while $V_{\perp}^{2\omega}$, $E_{\perp}^{2\omega}/\left(E_{\parallel}^{\omega}\right)^2$ and $\sigma^{(2)}$ show maxima. Such a contrast clearly shows that the variation of linear conductivity (or the resistance) with temperature is not the reason why we observed a peak in the nonlinear Hall response. Instead, it is **D** ($\propto E_{\perp}^{2\omega}/\left(E_{\parallel}^{\omega}\right)^2$) that results in a peak in the temperature dependence of nonlinear Hall voltage response. It is worth noting that the peak temperatures of $V_{\perp}^{2\omega}$, $E_{\perp}^{2\omega}/\left(E_{\parallel}^{\omega}\right)^2$, and $\sigma^{(2)}$ are different: while $E_{\perp}^{2\omega}/\left(E_{\parallel}^{\omega}\right)^2$ peaks at 206K, $V_{\perp}^{2\omega}$ and $\sigma^{(2)}$ show peaks at 250K and 193K respectively. This is because that $V_{\perp}^{2\omega}$ and $\sigma^{(2)}$ depend not only on **D** but also on $\sigma^{(1)}$; $V_{\perp}^{2\omega} \propto \mathbf{D}/[\sigma^{(1)}]^2$ and $\sigma^{(2)} = -\sigma^{(1)}\,E_{\perp}^{2\omega}/\left(E_{\parallel}^{\omega}\right)^2 \propto \mathbf{D}\sigma^{(1)}$. Given that $\sigma^{(1)}$ monotonically decreases with increasing temperature, the peak of $V_{\perp}^{2\omega}$ shifts to a higher temperature, whereas the peak of $\sigma^{(2)}$ shifts to a lower temperature.

10. Absence of NLHE in $SrMnSb_2$

The inversion symmetry breaking plays a critical role in generating the NLHE in $BaMnSb_2$. To demonstrate that, we also performed nonlinear transport measurements on a microscale Hall device fabricated using a lamellar crystal of $SrMnSb_2$, a sister compound of $BaMnSb_2$ (see Supplementary Fig. 14a). Like $BaMnSb_2$, $SrMnSb_2$ is also a Dirac material with gapped Dirac cones generated by Sb zig-zag chains.[11] However, unlike $BaMnSb_2$ which possesses a non-centrosymmetric structure, $SrMnSb_2$ crystalizes in a centrosymmetric *Pnma* orthorhombic structure.[11, 12] As expected, the $SrMnSb_2$ device shows an extremely small, fluctuated second-harmonic Hall response at room temperature, as shown in Supplementary Fig. 14b. We also performed a temperature scan from 50K to 350K, which does not reveal any enhancement with increasing temperature, indicating the absence of NLHE in $SrMnSb_2$.

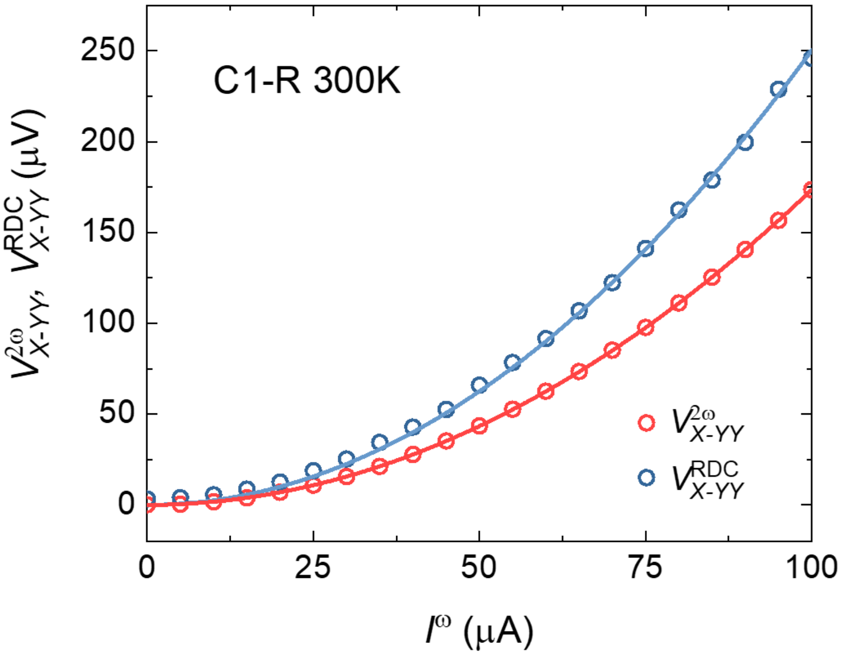

Supplementary Fig. 1 NLHE in device C1-R. Second harmonic and rectification Hall voltages as functions of driving ac at 300K for C1-R. Note that this set of data was measured a few weeks after the data presented in the main text (Fig. 3c & 3d) were collected. The discrepancy of $V_{X-YY}^{2\omega}$ between Fig. 3d and Fig. S1 can be attributed to sample degradation (the FIB-cut surface is susceptible to oxidation as discussed in Supplementary Note 8).

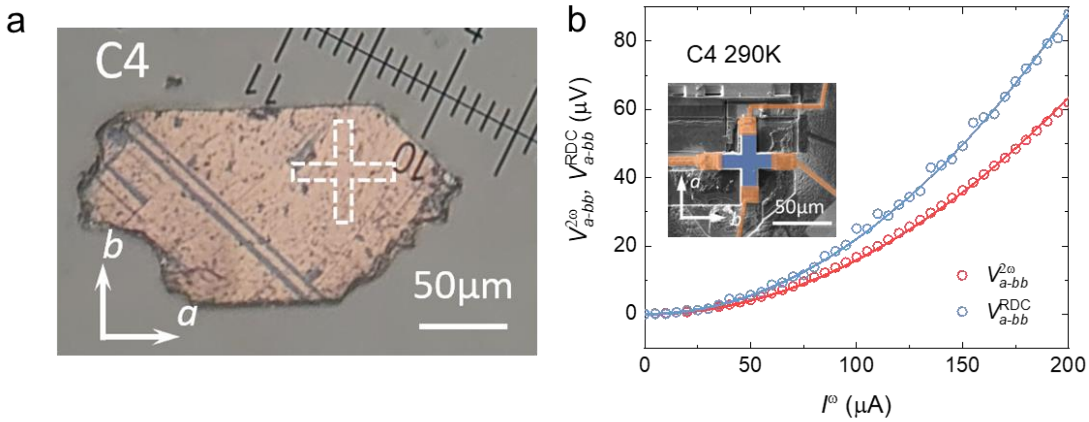

Supplementary Fig. 2 NLHE in device C4. (a) Optical image of the crystal which was used for fabricating device C4. The device position is marked by the white dashed lines. (b) Second harmonic and rectification Hall voltages as functions of driving ac at 290K for C4. Open circles and solid lines represent the experiment data and the quadratic fits, respectively. Inset: SEM image of the device.

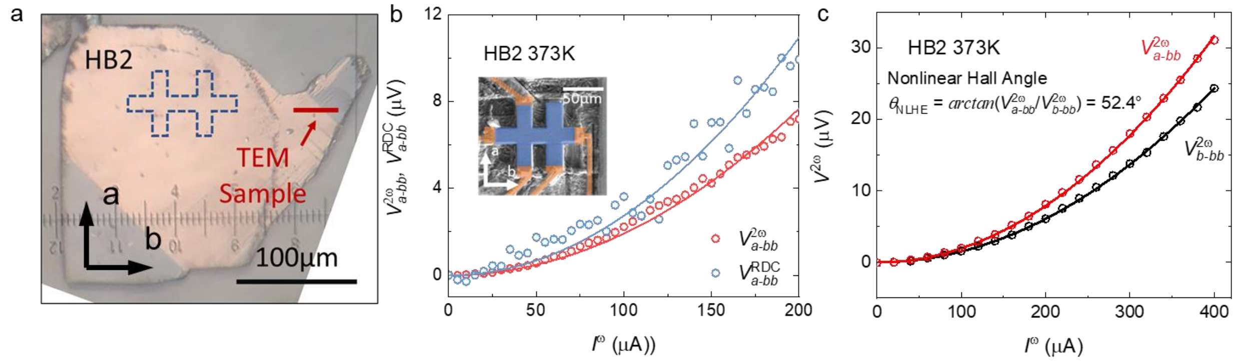

Supplementary Fig. 3 NLHE in device HB2. (a) Optical image of the crystal which was used for fabricating device HB2. The blue dashed lines mark the location of the HB2 device. To determine the crystallographic directions, we took a cross-section lamellar specimen across a domain boundary through FIB cutting for scanning transmission electron microscopy (STEM) analyses, as marked by the red solid line (see Supplementary Note 2). (b) Second harmonic and rectification Hall voltages as functions of driving ac at 373K for HB2. Inset: SEM image of the device. (c) The transverse and longitudinal second harmonic voltages as functions of the driving current. The nonlinear Hall angle, $\arctan(V^{2\omega}_{a-bb}/V^{2\omega}_{b-bb})$, derived from these data is 52.4°. Its large deviation from the expected nonlinear Hall angle of 90° could have two possible origins: (i) a longitudinal voltage component is mixed with the Hall voltage due to the voltage leads' misalignment; (ii) this sample involves striking inhomogeneity (i.e. the variation of domain structures and chemical potential throughout the sample).

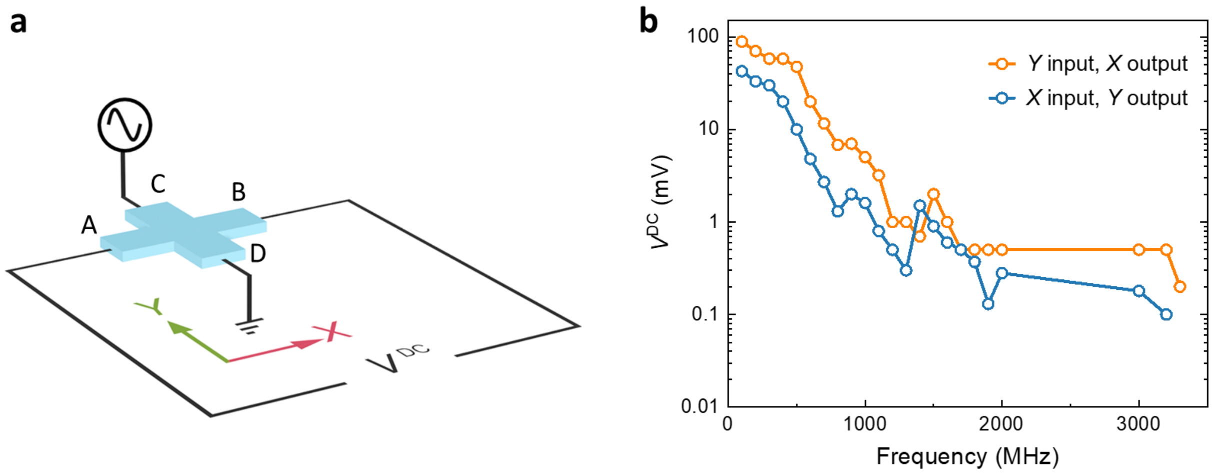

Supplementary Fig. 4 Rectification effect in the radio frequency range. (a) Schematic of the experiment setup. (b) dc output signal as a function of the input frequency in RF range.

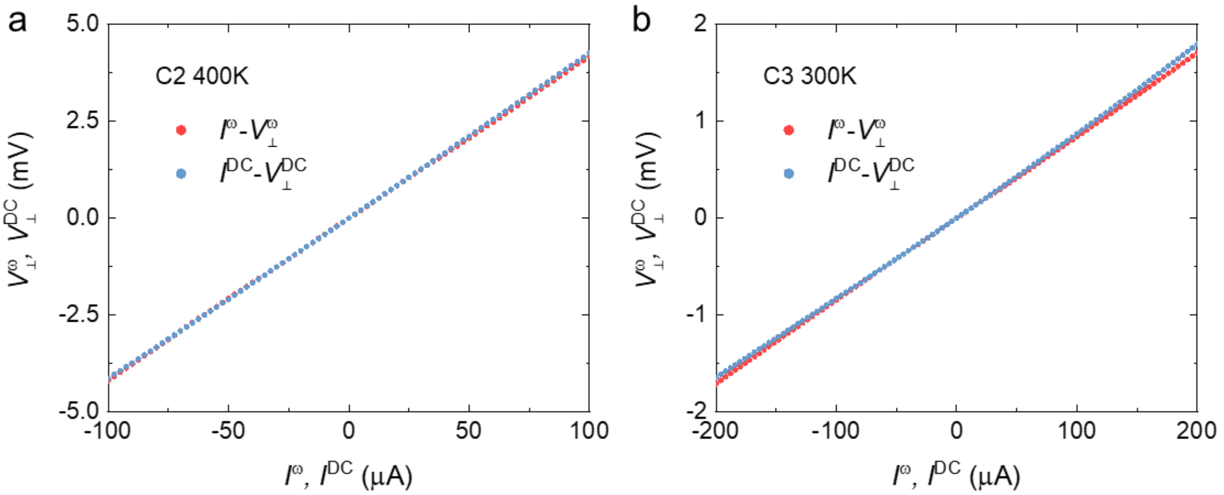

Supplementary Fig. 5 DC NLHE in C2 and C3. Comparison of the ac and dc *I*-*V* curves of C2 at 400K (a) and C3 at 300K (b). The dc *I*-*V* curves show nonlinear characteristics.

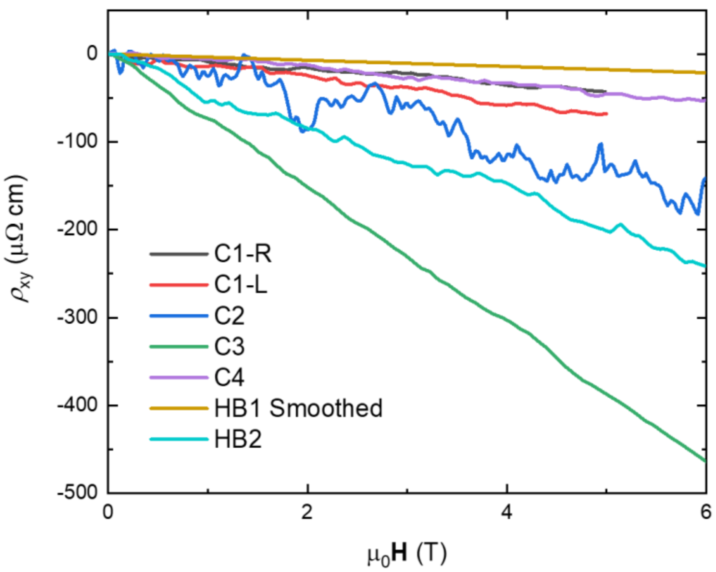

Supplementary Fig. 6 Normal Hall measurement of all 6 devices at room temperature.

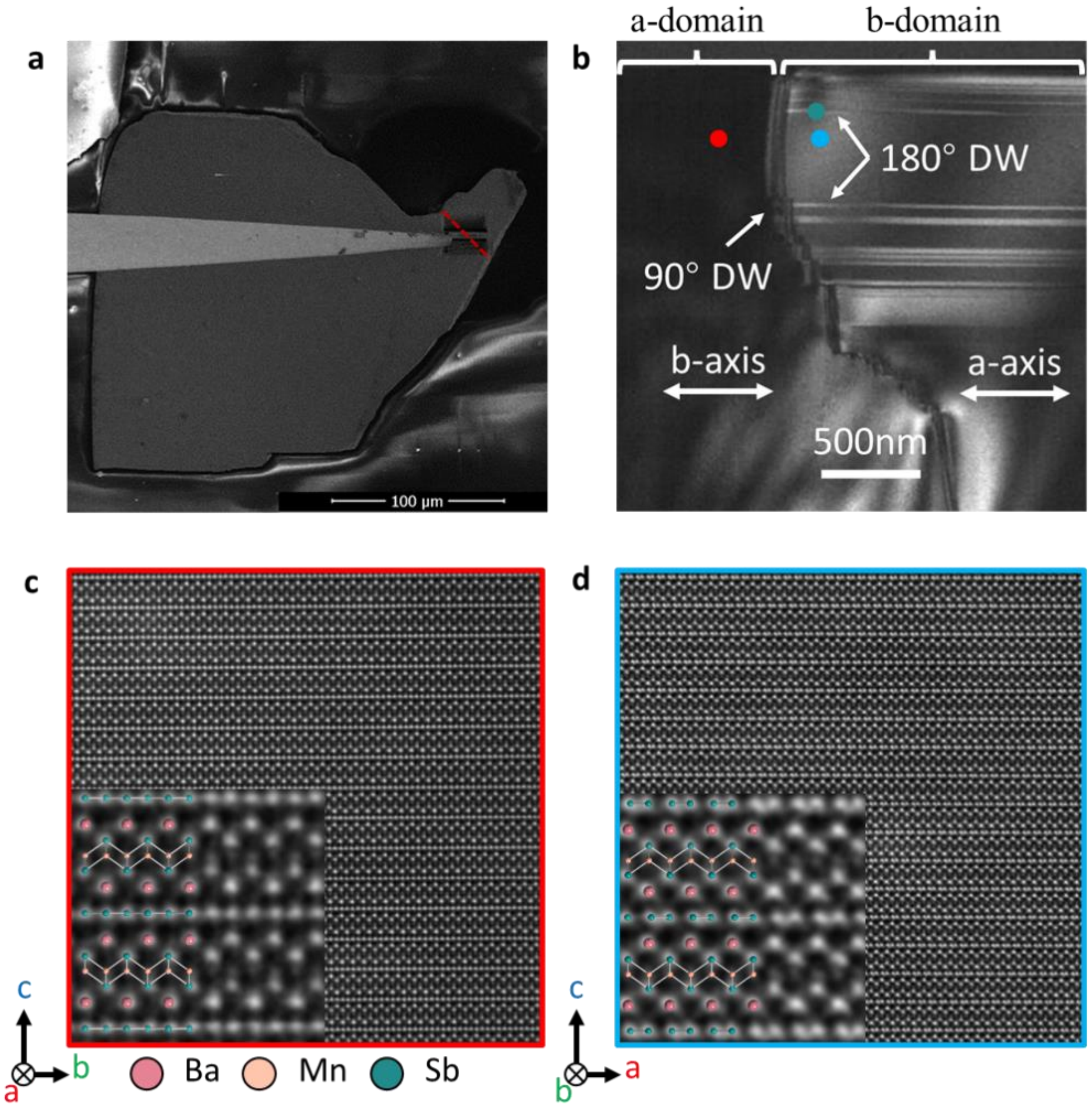

Supplementary Fig. 7 Domain analysis via STEM. (a)The SEM image showing the location of the STEM sample lifted out from the plate-like crystal where HB2 was extracted. A red dashed line marks the 90° domain wall. (b) The dark-field (DF-) TEM image taken with g = 020/200 reflection. The single vertical 90° and two horizontal 180° domain walls are marked with arrows. (c, d) The atomically resolved annular dark field (ADF-) STEM images taken from the a-domain and b-domain, respectively. The ADF-STEM images were taken at the positions marked by the red and cyan spots in (b). The insets are the magnified images with the $BaMnSb_2$ crystal model of the [100] and [010] axis superimposed.

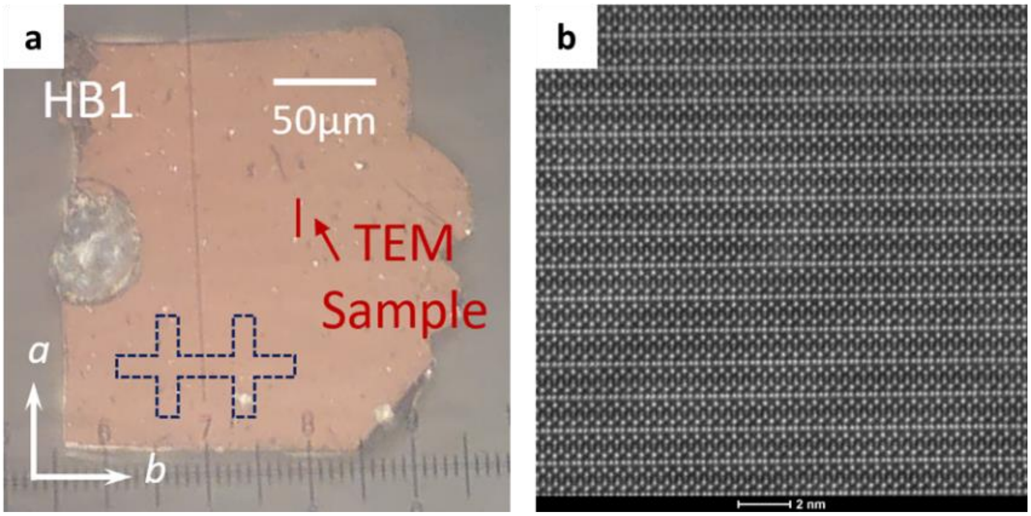

Supplementary Fig. 8 Crystallographic orientation determined by STEM. (a) The optical image of the lamellar crystal which was used for fabricating HB1. The blue lines mark the location of the HB1 device. The location where the STEM sample was extracted is marked by the red line. (b) The ADF-STEM image clearly shows the Sb 'dumbbell-shaped' pairs, which means the b-axis is vertical to the lamellar.

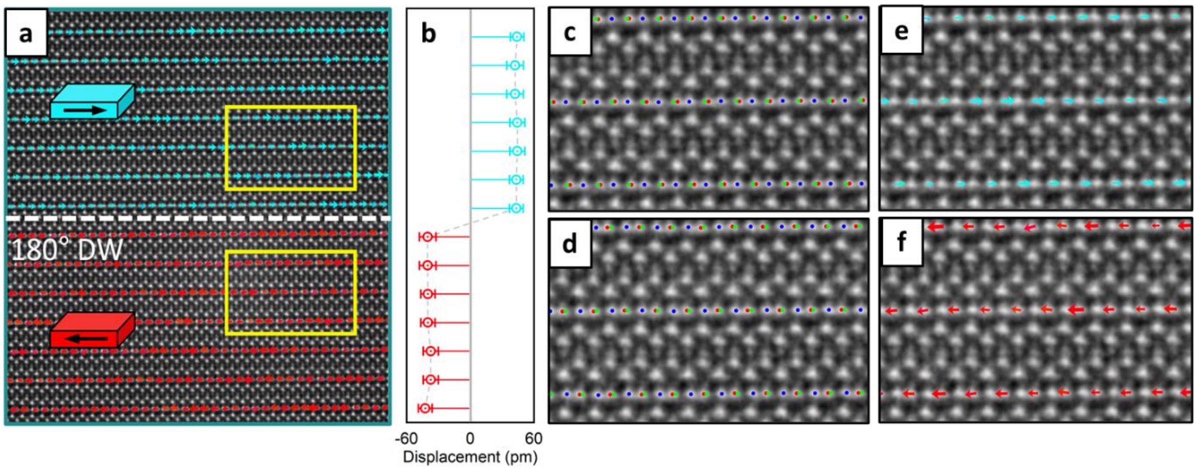

Supplementary Fig. 9 180° domain wall in $BaMnSb_2$. (a) The atomic displacement vector map superimposed on the atomically resolved ADF-STEM images near the 180° domain wall. The ADF-STEM image was taken at the position marked by the green spot in Fig. S7b. The 180° domain wall is marked with a white dashed line. (b) The displacement profile of each Sb layer shown in panel (a). The error bars are the standard deviations. (c, d) The magnified image in the yellow dashed box from (a), with the atomic positions of Sb in the zig-zag chain layer marked with colored spots. The Sb atomic columns near the unit cell center and edges are colored red and blue, and the reference center is colored green. (e, f) The atomic displacement vector measured from the same area in (c) and (d). The displacement is measured by comparing the position of the Sb atomic columns near the unit cell center (red spots) and the reference center (green spots).

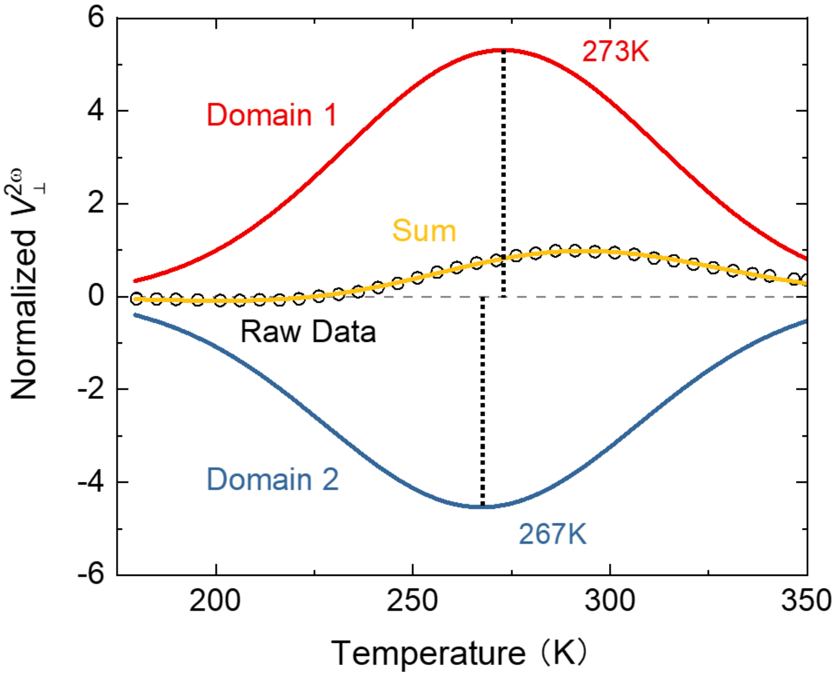

Supplementary Fig. 10 Two-peak fitting of the temperature-dependent NLH voltage measured in sample S4

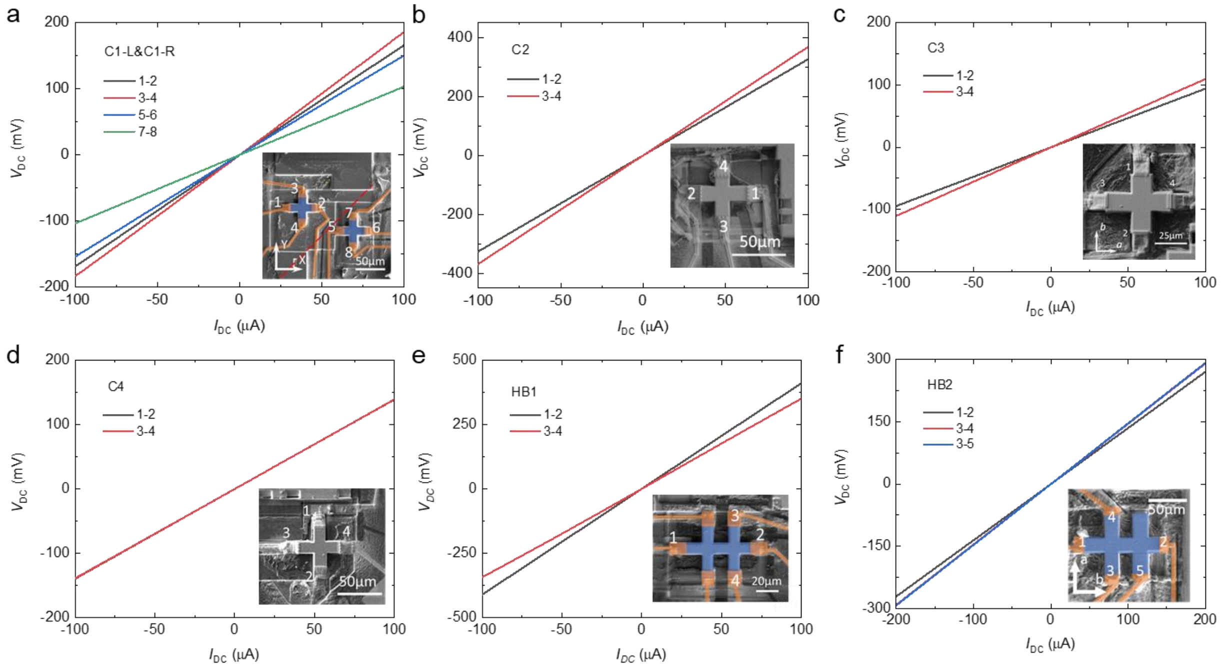

Supplementary Fig. 11 2-Probe DC I-V curve measurements at room temperature for all the seven samples used in this study. (a) C1-L&R, (b) C2, (c) C3, (d) C4, (e) HB1, and (f) HB2. All of them show nearly linear *I-V* responses, indicating there was no contact junction effect involved in these samples.

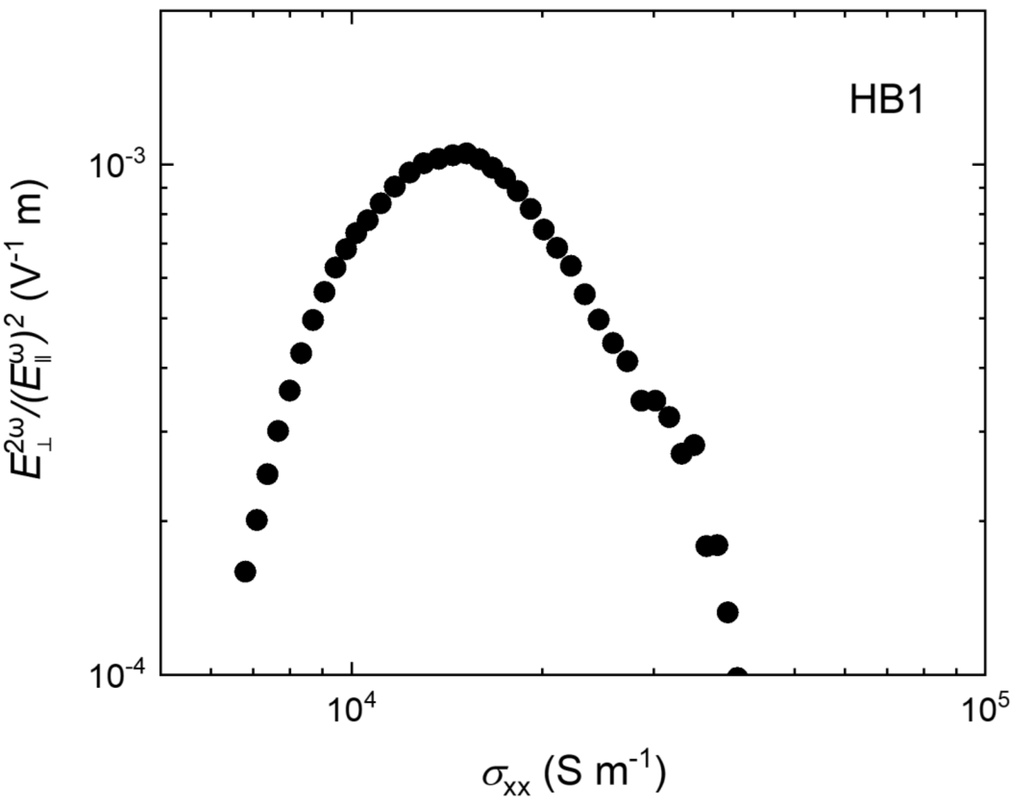

Supplementary Fig. 12 $E_\perp^{2\omega}/\left(E_\parallel^{\omega}\right)^2$ as a function of σ_{xx} in sample HB1

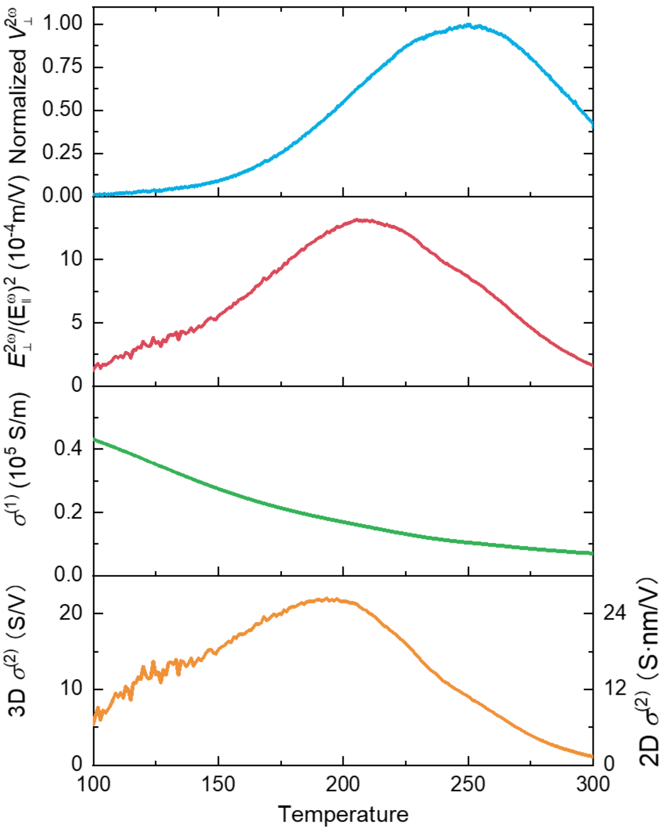

Supplementary Fig. 13 Temperature dependences of the normalized $V_\perp^{2\omega}$, $E_\perp^{2\omega}/\left(E_\parallel^{\omega}\right)^2$, $\sigma^{(1)}$ and $\sigma^{(2)}$.

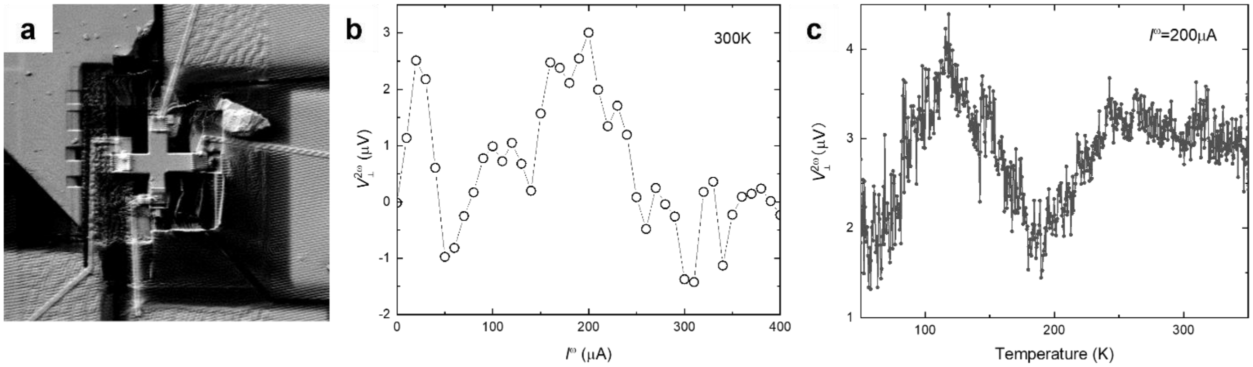

Supplementary Fig. 14 No NLHE was observed in $SrMnSb_2$ micro device. (a) SEM image of the cross-like $SrMnSb_2$ device. (b) $V_\perp^{2\omega}$ vs I^ω curve measured on the device in panel (a) at room temperature. (c) Temperature dependence of the second-harmonic Hall signal in $SrMnSb_2$ plate-like crystal, measured with an applied current of 200μA.

Supplementary Table 1 Carrier density n_e extracted from the Hall resistivity measurements for the samples used in this study.

Sample label	n_e(10K) (10^{19} cm^{-3})	n_e(RT) (10^{19} cm^{-3})	n_e(350K) (10^{19} cm^{-3})
C1-R	2.0	7.4	15
C2	2.4	2.4	2.4
C3	0.8	0.8	1.3
C4	3.2	8.2	20 (325k)
HB1	4.4	17	
HB2		2.4	3.0

Reference

1. Liu JY, *et al.* Spin-valley locking and bulk quantum Hall effect in a noncentrosymmetric Dirac semimetal $BaMnSb_2$. *Nature Communications* **12**, 4062 (2021).

2. Miao L, Chmielewski A, Mukherjee D, Alem N. Picometer-Precision Atomic Position Tracking through Electron Microscopy. *JoVE*, e62164 (2021).

3. Ma Q, *et al.* Observation of the nonlinear Hall effect under time-reversal-symmetric conditions. *Nature* **565**, 337-342 (2019).

4. Sakai H, *et al.* Bulk quantum Hall effect of spin-valley coupled Dirac fermions in the polar antiferromagnet $BaMnSb_2$. *Physical Review B* **101**, 081104 (2020).

5. Moll PJW. Focused Ion Beam Microstructuring of Quantum Matter. *Annual Review of Condensed Matter Physics* **9**, 147-162 (2018).

6. Kumar D, *et al.* Room-temperature nonlinear Hall effect and wireless radiofrequency rectification in Weyl semimetal $TaIrTe_4$. *Nature Nanotechnology* **16**, 421-425 (2021).

7. Du ZZ, Wang CM, Li S, Lu H-Z, Xie XC. Disorder-induced nonlinear Hall effect with time-reversal symmetry. *Nature Communications* **10**, 3047 (2019).

8. Kang K, Li T, Sohn E, Shan J, Mak KF. Nonlinear anomalous Hall effect in few-layer WTe_2. *Nature Materials* **18**, 324-328 (2019).

9. Huang M, *et al.* Giant nonlinear Hall effect in twisted WSe_2. *arXiv*, (2020).

10. He P, *et al.* Graphene moiré superlattices with giant quantum nonlinearity of chiral Bloch electrons. *Nature Nanotechnology* **17**, 378-383 (2022).

11. Liu JY, *et al.* A magnetic topological semimetal $Sr_{1-y}Mn_{1-z}Sb_2$ (y, z < 0.1). *Nature Materials* **16**, 905-910 (2017).

12. Brechtel E, Cordier G, Schäfer H. Neue ternäre erdalkali-übergangselement-pnictide. *Journal of the Less Common Metals* **79**, 131-138 (1981).
